# Provision of straw by a foraging tower –effect on tail biting in weaners and fattening pigs

**DOI:** 10.1186/s40813-017-0052-7

**Published:** 2017-03-16

**Authors:** Carolin Holling, Elisabeth grosse Beilage, Beatriz Vidondo, Christina Nathues

**Affiliations:** 1 University of Veterinary Medicine Hannover, Field Station for Epidemiology, Büscheler Str. 9, D-49456 Bakum, Germany; 2 Veterinary Public Health Institute, Vetsuisse Faculty, Schwarzenburgstrasse 155, CH-3097 Liebefeld, BE Switzerland

**Keywords:** Environmental enrichment, Welfare, Exploratory behaviour, Ammonia

## Abstract

**Background:**

Straw is one of the most effective rooting materials to reduce tail biting in pigs. A so-called foraging-tower (FT) provides only small quantities of straw compatible with liquid manure systems. The focus of the present study was on the effect of providing straw by FT in order to prevent tail biting in tail docked pigs. Four consecutive batches of 160 pigs, randomly divided into a straw (SG) and a control group (CG) were followed up from weaning to slaughter.

**Results:**

Tail wounds (Score ≥ 2) were detected in 104 out of 12,032 single observations (SG n = 48; CG *n* = 56) in 9 pens (SG *n* = 4/32; CG *n* = 5/32) mainly focused on the fattening period of batch 2 due to a failure in the ventilation system. No significant differences concerning the distribution of Score ≥ 2 in pens of the SG and CG could be identified. Bite marks (Score 1) were documented in 395 observations at animal level (SG *n* = 197, CG *n* = 198) in all batches. In the nursery period, the air velocity significantly increased the chance that at least one pig per pen and week showed a tail lesion score ≥1 (*p* = 0.024). In the fattening period ammonia concentration was positively associated with tail lesions (*p* = 0.007).

The investigation of blood samples revealed infections with *Mycoplasma hyopneumoniae* in all batches and a circulation of Porcine Reproductive and Respiratory Syndrome Virus (NA-vaccine strain) and Porcine Circovirus Type 2 in two batches each. The average daily straw consumption was 3.5 g/pig (standard deviation (SD) = 1.1) during the rearing period and 31.9 g/pig (SD = 7.7) during the fattening period.

**Conclusion:**

Due to the low prevalence of tail biting in all batches the effect of the FT tower could not be evaluated conclusively. The operation of the FT with an average daily straw consumption of 3.5 g/pig (SD = 1.1) during the rearing period and 31.9 g/pig (SD = 7.7) during the fattening period did not affect the weight gain. Exploratory behaviour seems to cause bite marks (score 1), which do not necessarily result in tail biting. The main outbreak of tail biting was probably triggered by a failure of the ventilation system, which resulted in a number of climatic and air quality changes including higher ammonia concentrations and sudden temperature changes.

**Electronic supplementary material:**

The online version of this article (doi:10.1186/s40813-017-0052-7) contains supplementary material, which is available to authorized users.

## Background

Tail biting is an abnormal behaviour in the domestic pig causing reduced animal welfare and economic losses in pig production worldwide [1]. Despite the prohibition according to Council Directive 2008/120/EC [2], most pigs in conventional pig production in the EU are tail docked [3, 4]. Only when there is evidence that pigs have previously been injured by tail biting and on condition that inadequate environmental conditions and management systems have been addressed previously, is tail docking exceptionally allowed. The European Food Safety Authority (EFSA) estimates a prevalence of tail biting in docked pigs around 3%, while the prevalence in undocked pigs in Finland, Sweden and Norway, where tail docking is totally banned, is assumed to be between 6 and 10% and as high as up to 30% [3].

Although tail docking is effective in reducing the prevalence of tail biting, it can neither totally solve the problem [5, 6], nor is it consistent with animal welfare [3, 7]. However, victims of tail biting suffer from acute pain, have an increased risk of infections and a reduced weight gain. As a consequence, tail biting is not compatible with welfare and it is in the interest of pig producers to avoid economical losses due to tail biting [1, 8]. Nevertheless, the prevention of tail biting should focus on identifying and eliminating predisposing, possibly interacting risk factors. These include, for example, a lack of manipulable enrichment materials, a high stocking density, poor air quality, poor health, a reduced feed and water quality, restricted feeding and drinking systems as well as genetics [9, 10]. By fulfilling the pigs’ natural exploratory behaviour, which consists of rooting, sniffling, biting and chewing various digestible and indigestible items, the provision of rooting material can reduce the risk of tail biting [11]. On this account permanent access to a sufficient quantity of material is also regulated by law to increase animal welfare (Council Directive 2008/120/EC) [2]. In comparison to most enrichment objects (e. g., chains, rubber hoses, balls) rooting materials like straw or wood shavings prevent tail biting more effectively [11, 12]. Assuming a behavioural synchronisation of pigs even the accessibility of materials or objects could play an important role in avoiding aggression due to competitive behaviour [13].

However, in Western Europe most current housing systems have fully slatted floors and liquid manure systems, which could be blocked by offering large amounts of straw [12, 14]. A so-called foraging-tower (FT) provides only small quantities of straw compatible with liquid manure systems. The focus of this study was on the effect of providing straw by FT in order to prevent tail biting in tail docked pigs.

## Methods

The study was conducted from June 2013 to August 2014 in a conventional farrow to finish herd in Germany with a history of tail biting in fattening pigs for several years. In the past, the prevalence of tail biting had been estimated as being up to 15% in affected batches, although the tails had been docked.

### Animals, housing and management

The piglets, Landrace x Large White x Pietrain crossbreds, were tail docked within the first three days of life and male pigs were castrated within the first week of life. During the suckling period piglets had been vaccinated against Porcine Circovirus Type 2 (PCV2) (Ingelvac CircoFLEX, Boehringer Ingelheim Vetmedica, Inc., D-55216 Ingelheim/Rhein, Germany) and *Mycoplasma hyopneumoniae* (MH) (Porcilis® M Hyo, Intervet Deutschland GmbH, D-85701 Unterschleissheim, Germany). The sows on the farm were regularly vaccinated against Porcine Reproductive and Respiratory Syndrome (PRRS) (Ingelvac PRRS® MLV, Boehringer IngelheimVetmedica, Inc.), Erysipelas and Parvovirus (Porcilis® Ery + Parvo, Intervet Deutschland GmbH) as well as Swine Influenza Virus (SIV) (RESPIPORC FLU3, IDT Biologika GmbH, D-06861 Dessau-Rosslau, Germany). After a suckling period of 26 days, all weaners (*n* = 480) were sorted by weight (low, medium, high) in three groups due to the farmer’s routine management. Each weight group was housed in one of the units of the rearing barn containing eight pens (3.2 × 2.5 m) with 20 pigs each. Only the weaners of the medium weight group (*n* = 160) were included in the study.

The floor was fully slatted, consisting of one third concrete and two thirds plastic. The pigs were fed automatically ad libitum with commercial feed (Table 1) by a wet/dry swing feeder (AP Company, DK-7570 Vemb, Denmark) in each pen. Water was available ad libitum via two drinking nipples per pen.

**Table 1:** Commercial feeds used in the study from wean to finish

Type of feed	Weight of pigs^a^ (kg)	Form of feed	ME^b^/kg	Crude protein (%)	Lysine (%)	Crude fibre (%)	Crude oils and fats (%)
complete feed for piglets	5.5—6.5	small pellets	16.00	23.00	1.65	2.20	8.50
complete feed for piglets	6.5—10	pellets (2.2 mm)	14.00	20.00	1.40	3.30	3.30
complete feed for piglets	10—30	pellets (3.0 mm)	14.00	20.00	1.32	3.50	4.00
complete feed I for fattening pigs	30—50	liquid	13.20	17.00	1.05	4.20	3.70
complete feed II for fattening pigs	50—120	liquid	13.40	14.00	0.95	3.70	3.40

^a^To avoid sudden changes in diet, feed was blended with the following feed for at least one week

^b^metabolizable energy

At a weight of approximately 28 to 30 kg pigs were vaccinated against SIV (RESPIPORC FLU3, IDT Biologika GmbH) and moved to the fattening barn in which two equal units, containing 16 pens each, were used for the study. Ten pigs were kept in each pen (3.2 × 2.5 m). All pens were equipped with fully slatted concrete floor and a long trough (length: 3.2 m; animal: feeding place ratio 1:1). The pigs of two adjoining pens were fed restrictively eight times a day with commercial feed (Table 1) by one feeding valve of a liquid feeding system. Water was available ad libitum via one drinking nipple per pen. Artificial light was switched on from 6:30 h to 20:00 h. The ventilation system consists of spray cooling channels, an overhead extraction system and a heating system operated with biogas.

### Foraging tower and dummy

**Fig. 1:**
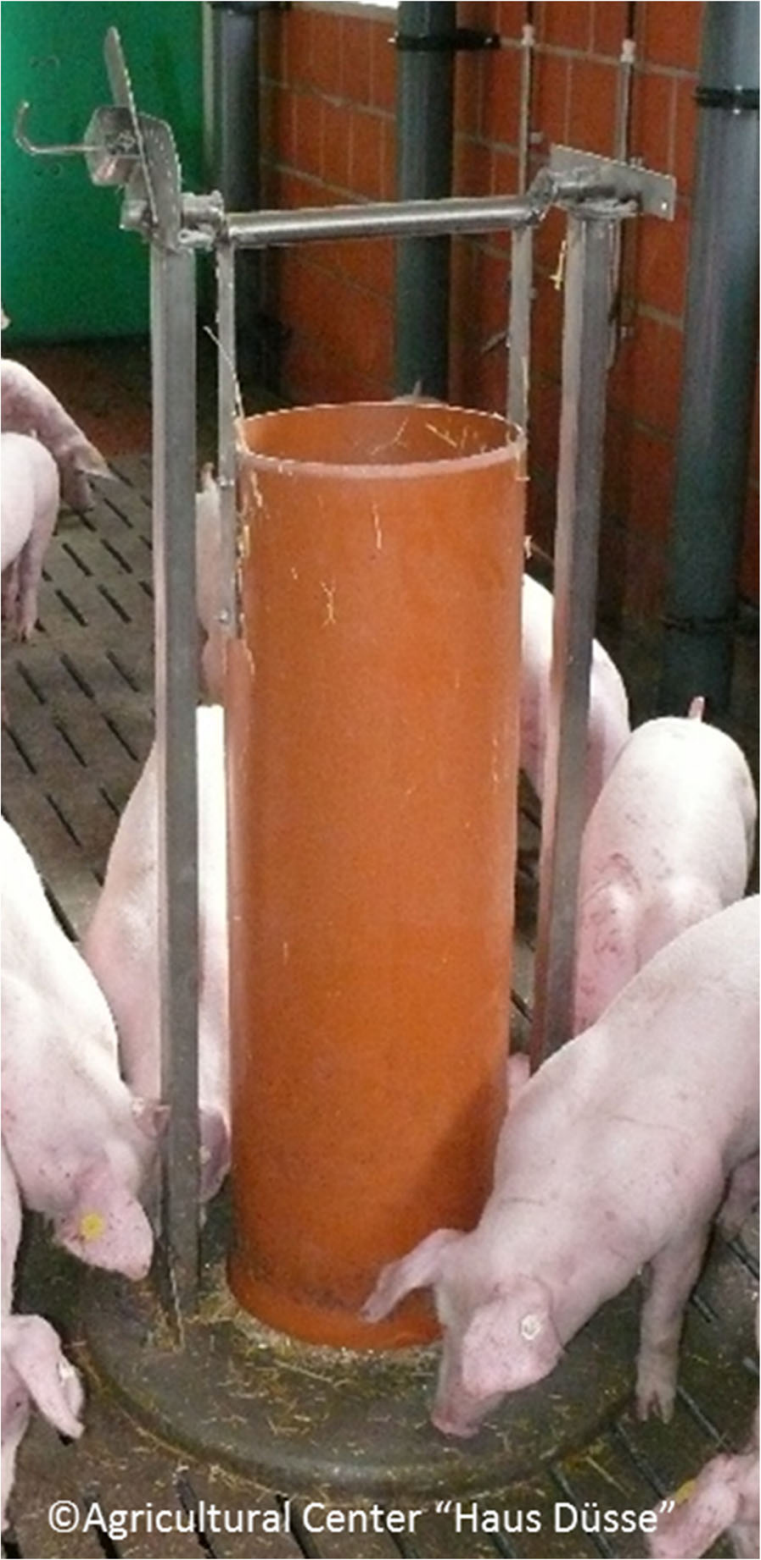
foraging tower

The foraging tower (Fig. 1) was developed by the Agricultural Center “Haus Düsse” (D-59505 Bad Sassendorf, Germany) for fattening pigs and consists of a movable plastic tube used as a container (height 130 cm, diameter: 31 cm) and a round concrete base (diameter 70 cm) on which it is fixed. The container was permanently filled with short-chopped wheat straw (length: 5–7 cm) of high quality. The pigs nudge the material from a small adjustable gap (adjusted size in this study: 2.5—4.0 cm) between the container and the concrete base. For usage during the rearing period smaller foraging towers had been manufactured (plastic tube: height 110 cm, diameter 20 cm, concrete base: diameter 65 cm). Prior to the experiment the pens for the straw group (SG) were randomly selected and a foraging tower was installed. The remaining pens for the control group (CG) were equipped with an equally sized dummy consisting of a concrete base and a plastic tube, which was neither movable nor filled with straw.

### Experimental design

**Fig. 2:**
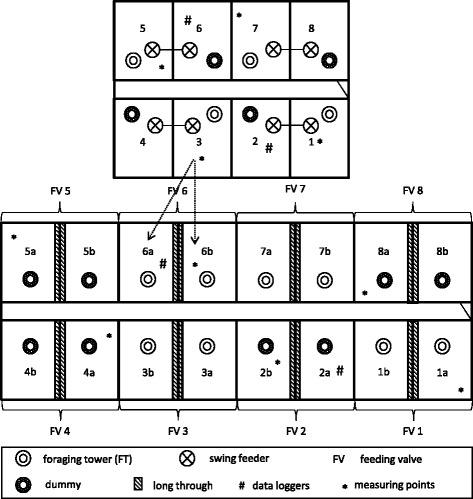
Illustration of unit design in the rearing (above) and fattening barn (below) used in this study (not true to scale). The *arrows* show an option of dividing pigs of one rearing pen (20 pigs) into two fattening pens (2 × 10 pigs)

In total, 640 pigs in 32 rearing pens and 64 fattening pens were followed up from weaning to slaughter in four consecutive batches of 160 pigs each. At weaning 80 castrated male pigs and 80 female pigs of the medium weight group were randomly selected for the straw (SG) or control (CG) group, individually marked with a numbered ear tag and allocated to eight pens (20 pigs per pen) separated by gender. For the fattening period the pigs of each pen were randomly divided into two groups and placed in two neighbouring pens, fed by one feeding valve, in the fattening barn (16 pens, 10 pigs per pen). The unit design in the rearing and fattening barn is shown in Fig. 2.

### Data collection

At the beginning and at the end of the rearing and fattening period the weight of the pigs per pen was measured. Once a week the tails of all pigs were scored by the same observer using the parameters “tail lesion” and “blood freshness” (Table 2, modified from Zonderland et al.) [12] and documented for each pen. Scores 2, 3, 4 and 5 were defined as different stages of tail biting. Pigs with tail wounds ≥ Score 2 were individually identified by their ear tag number and recorded. In cases of tail biting the scoring interval was reduced to every second day and slowly increased, if no fresh bleeding tail wounds had occurred any more. The scoring was stopped, when the farmer delivered the first group of pigs to slaughter.

**Table 2:** Scores for the parameters tail lesion and blood freshness (modified from Zonderland et al., [12])

Parameter	Score	Description
Tail lesion	0	No tail lesion visible
	1	Little lesions/bite marks are visible (size of a pinhead)
	2	Clearly visible wound ≤ cross section of the tail
	3	Clearly visible wound ≥ cross section of the tail without signs of inflammation (redness, swelling, heat)
	4	Clearly visible wound ≥ cross section of the tail with mild signs of inflammation (redness, swelling, heat)
	5	Clearly visible wound ≥ cross section of the tail with severe signs of inflammation (redness, swelling, heat)
Blood freshness	0	No blood visible
	1	Old dried black blood in the form of a scab
	2	Sticky dark red blood, mainly half a day to a day old
	3	Fresh bleeding wound

At the same time the ammonia content and the air velocity were measured with a gas detector (Draeger Pac® 7000, Draegerwerk AG & Co. KGaA, D-23558 Luebeck, Germany) and a hot-wire anemometer (testo 491, Testo AG, D-79853 Lenzkirch, Germany) at four (rearing unit) or six (fattening unit) predefined locations, respectively (Fig. 2). The air temperature was recorded continuously by two data loggers (testo 175 T1, Testo AG, D-79853 Lenzkirch, Germany) per unit.

Blood samples from 22 randomly selected pigs were taken at the beginning and at the end of the fattening period of each batch (paired serum samples) and tested by enzyme linked immunosorbent assay (ELISA) for antibodies against PRRSV (IDEXX PRRS X3®, IDEXX Switzerland AG, CH-3097 Liebefeld-Bern, Switzerland) and MH (IDEXX M. hyo®, IDEXX Laboratories, Inc., Westbrook, Maine, 04092 USA). PCR’s for PRRSV (EZ-PRRSV™ MPX 4.0 RT-PCR®, Tetracore Inc., Rockville, MD 20850, USA) and PCV2 (according to Brunborg et al. [15]) were performed in eight pool samples with a maximum of three samples per pool from the beginning and eight pool samples from the end of the fattening period per batch.

The feed consumption per feeding valve was recorded by the feeding computer (HOWEMA Gerätebau GmbH & Co. KG, D-49429 Visbek, Gemany). Straw consumption was recorded by the farmer documenting the number of previously weighed 2.5 kg-bags of straw per FT he used for the daily replenishment. The remaining straw of the last bag per FT and the amount of straw in the FT were weighed again and subtracted at the end of each period.

### Statistical analysis

Data storage and management was done in Microsoft Excel® (Version 2010, Microsoft Corporation, Redmond, Washington, US) and statistical analyses were performed in R software version 3.3.1 (R Core Team (2016)). The unit of observation was the pen in a given week (“pen-week”). Scorings routinely performed once a week and the scorings that were additionally carried out in case of tail biting were summarized using the highest scores per week and animal. After that, scorings yielded a number of animals per score (0–5) for each pen. For statistical analyses, this information was further combined in a summary score per pen-week, in which each tail lesion score was multiplied by the number of animals exhibiting this score, and the sum of these values taken. If, e.g., in a pen consisting of 20 animals, in a given week two pigs showed tail lesion score 2 and one pig showed tail lesion score 3, the summary score of this pen-week was 2 * 2 + 1 * 3 = 7. Furthermore, pen-week was dichotomized (at least one pig with score ≥1 = positive vs. no pig with score ≥1 = negative).

To assess the effect of treatment (the foraging tower) on the occurrence of tail biting, a series of mixed effects models were calculated for nursery and fattening period separately, using R package ‘lme4’. Preliminary analyses included tests for normality of numerical variables with Kolmogorov-Smirnov-Test and linearity with the outcome variable. All variables were tested for associations with the outcome or correlations between variables, using Chi^2^-Tests, Mann-Whitney *U*-Test and Spearman’s rank correlation coefficient, depending on the scale of the variables. In the case of a correlation coefficient of *r* > 0.6, the more biologically meaningful variable was used in the multivariable model. Calculated *p*-values of less than 0.05 were considered statistically significant.

Due to the low occurrence of tail biting over the whole period of observation, with many pen-weeks where no pigs exhibited tail lesion scores above 0, it was not possible to fit a model to all observations with any discrete-scale outcome variable (e.g., the summary score or the number of pigs with score ≥ 2 per pen-week). For this reason, a two-step approach was chosen: In a first step, a generalized linear mixed-effects model including all observations was calculated with dichotomized pen-week as outcome and the treatment group as main effect. To account for the climatic conditions, also the ammonia concentration, the air velocity, the highest day/night temperature range and the average temperature in that week were considered as fixed effects. These temperature variables were chosen because they were deemed to be the most comprehensive and least correlated ones (as opposed to minimum and maximum temperature). Furthermore, to account for the fact that observations in the same pens were made at different times, the week was also included as fixed effect. Lastly, to account for the hierarchical structure of the data of pen-week being nested in pen and pen being nested in batch, and thus to account for the additional influence of pen and batch, a random effect for pen nested in batch was included (random intercept model). In a second step, a linear mixed-effects model was calculated only with the positive pen-weeks (summary score > 0), using the discrete-scale summary score but otherwise with the same set-up as described for the first model. To meet the assumptions necessary for a linear model (i.e., the normality and linearity, tested visually with quantile-quantile-plots), the summary score was log-transformed.

The modelling process consisted of 1) testing each explanatory variable (group, ammonia concentration, air velocity, average temperature and day/night temperature) individually with pen nested in batch as random effect and correcting for week as fixed effect; 2) running the multivariable model with all four explanatory variables; 3) manual backward selection based on the highest *p*-value, until all explanatory variables retained in the final model had significant *p*-values.

Furthermore, the effect of treatment on the parameters feed consumption and average weight gain per day per fattening pig was assessed in two linear mixed models with treatment group as fixed effect and batch as random effect (random intercept models). The association between the quantity of straw consumption and feed consumption (outcome variable) in the fattening period within the TG was assessed in a linear mixed model with straw consumption as fixed effect and batch as random effect (random intercept model). Lastly, for nursery pigs, the same model as in fattening pigs was calculated for weight gain, whereas data on feed consumption were not available for the nursery period.

## Results

**Fig. 3:**
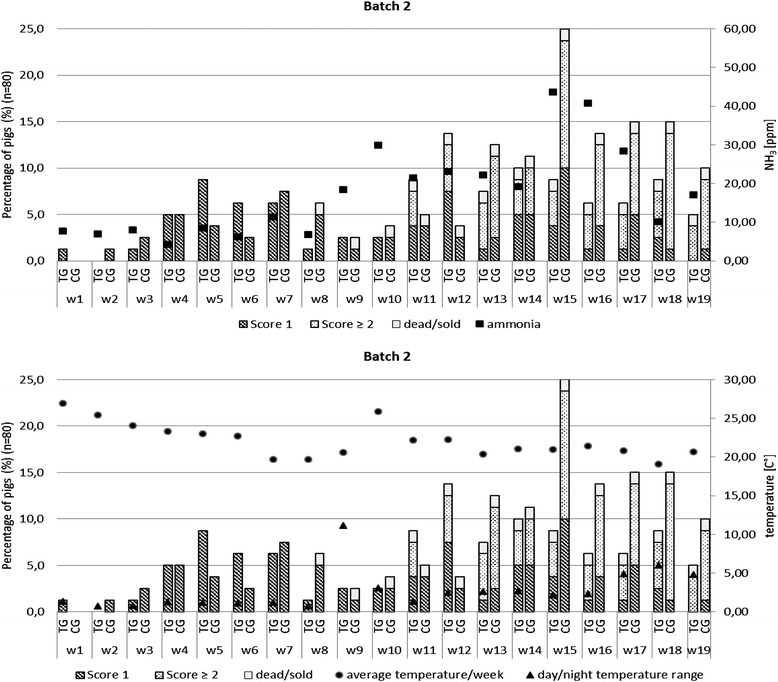
**a**: Bite marks (score 1) and tail wounds (score ≥ 2) related to the ammonia content (NH_3)_ in the units of batch 2 (w = week). **b**: Bite marks (score 1) and tail wounds (score ≥ 2) related to the average temperature and the highest day/night temperature range per week in the unit of batch 2 (w = week)

In total, the occurrence of tail lesions was analyzed in 12,032 single observations at animal level (SG *n* = 6016, CG *n* = 6016) and 976 (SG *n* = 488, CG *n* = 488) observations at pen-week level. Tail wounds (score ≥ 2) were detected in 104 single observations (SG *n* = 48; CG *n* = 56) in different 9 pens (SG *n* = 4/32; CG *n* = 5/32). The number of affected pigs per pen and the duration of tail biting are shown in Table 3. The duration was calculated as the number of consecutive weeks in which at least one pig per pen with a fresh bleeding tail wound was present. The occurrence of tail biting was mainly focused on four pens with 15 affected pigs in batch 2 and one pen during the rearing period of batch 4. Bite marks (score 1) were documented in 395 observations at animal level (SG *n* = 197, CG *n* = 198). The proportions of pigs with tail wounds (score ≥ 2) and bite marks (score 1) in all pens per week in the SG and CG are shown in Fig. 3 a and b for batch 2 and in the Additional files 1 a, b, 2 and 3 a, b for batch 1, 3 and 4.

**Table 3:** Number of pigs affected by tail biting per batch, pen and period and the duration of tail biting per pen

Batch	Pen	Straw group (affected pigs)	Control group (affected pigs)	Period	Duration of tail biting^b^ (weeks)
1	105	1	–	rearing	1
2	205a	–	1	fattening	1
2	205b	–	6	fattening	7
2	207b	4	–	fattening	9
2	208a	–	4	fattening	5
3	305b	–	2	fattening	2
4	405	5	–	rearing	4
4	402a		1	fattening	2
4	406a	1^a^	–	fattening	1

^a^The pig was one of the five affected pigs from pen 405 during rearing period

^b^Number of consecutive weeks in which at least one pig with a fresh bleeding was present (Score 3 for blood freshness)

**Fig. 4:**
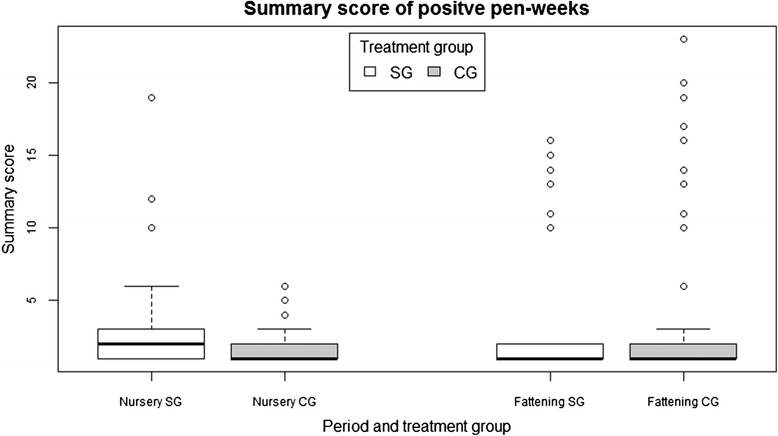
Boxplot of the summary score in positive pen-weeks, grouped by period and treatment group

In the nursery, 58 of 108 pen-weeks (53.7%) in the SG had at least one pig with score ≥ 1 (= positive pen-week), compared to 52 (58.1%) in the CG. A score of 5 was not seen in any pen-weeks in the nursery, whereas in two pen-weeks the highest score observed in a pig was 4, one pen-week had a highest score of 3 and another one a highest score of 2 (all in the SG).

In the fattening period, the number of positive pen-weeks was 53 of 256 (20.7%) in the SG and 67 (26.2%) in the CG. There were 13 pen-weeks (SG = 6, CG = 7) in which the highest score observed in an animal was 5; in five pen-weeks (SG = 3, CG = 2), the highest score was 4; in 2 pen-weeks (all CG), the highest score was 3 and in 5 pen-weeks (SG = 1, CG = 4), the highest score was 2. The summary scores﻿ in positive pen-weeks in the SG and CG are shown in Fig. 4 for each period.

The severest outbreak of tail biting with the highest number of affected pigs (score ≥ 2) was observed in the fattening period of batch 2. Due to a temporary failure in the ventilation system during the fattening period of the second batch the temperature loggers recorded a sudden rise in temperature of 11.5° Celsius [°C] within 27 h (days 62 and 63). The average temperature in the following week (week 10: 25.9 °C) was 5.3 °C higher than in the previous week (week 9: 20.6 °C). The measured ammonia concentrations were 18.5 ppm on day 62 and 29.8 ppm on day 69. During the following weeks ammonia contents increased up to 43.7 ppm. Tail biting started on day 67. Otherwise, no clear temporal patterns in the proportion of pigs showing bite marks (score 1) and tail wounds (score ≥ 2) over the course of the rearing and fattening period could be observed (Fig. 3 a and b and Additional files 1 a, b, 2 and 3 a, b).

The results of the mixed-effect models to assess the effect of treatment and climate variables on the occurrence of tail biting, accounting for the time, pen and batch, are indicated in Tables 4, 5, 6 and 7. For the treatment, no statistically significant effect could be identified in any of the models. In the nursery period, only the air velocity significantly increased the chance that a pen-week had at least one pig with score ≥1 in the final model (*p* = 0.024). Among the positive pen-weeks, the day/night temperature range showed a positive (*p* = 0.014) and the average temperature a negative (*p* = 0.003) association with the summary score. In the fattening period, in the final model only the day/night temperature range (*p* = 0.027) had an effect on whether the pen-week was positive or not, whereas ammonia concentration was positively associated with the summary score (*p* = 0.007).

**Table 4:** Results of the generalized linear mixed-effects model for the nursery period with dichotomized pen-week (at least one pig with score ≥1 vs. no pig with score ≥1) as outcome variable and pen nested in batch as random effect (random intercept): results of models for each individual explanatory variable, the full model and the final model

Nursery	Individual models		Full model		Final model^a^	
Fixed effect	Estimate	*P*-value	Estimate	*P*-value	Estimate	*P*-value
Week	−0.077	0.045	−0.040	0.679	−0.059	0.529
SG (CG = baseline)	0.331	0.590	0.330	0.539		
day/night temperature range	0.204	0.063	0.150	0.223		
average temperature	−0.167	0.245	model did not converge with this variable included		model did not converge with this variable included	
ammonia concentration	−0.024	0.521	−0.065	0.112		
air velocity	27.265	0.024	26.233	0.055	27.265	0.024

^a^random effect batch:pen (Intercept): variance 1.927

**Table 5:** Results of the linear mixed-effects model for the nursery period with log-transformed summary score per pen-week as outcome variable and pen nested in batch as random effect (random intercept): results of the models for each individual explanatory variable, the full model and the final model

Nursery	Individual models		Full model		Final model^a^	
Fixed effect	Estimate	*P*-value	Estimate	*P*-value	Estimate	*P*-value
Week	0.072	0.001	−0.078	0.070	−0.041	0.001
SG (CG = baseline)	0.259	0.141	−0.322	0.067		
day/night temperature range	−0.010	0.698	0.157	0.005	0.135	0.014
average temperature	−0.075	0.037	−0.269	0.001	−0.226	0.003
ammonia concentration	−0.005	0.682	0.007	0.637		
air velocity	−0.321	0.936	3.895	0.402		

^a^random effect batch:pen (Intercept): variance 0.1271, residual variance 0.2643

**Table 6:** Results of the generalized linear mixed-effects model for the fattening period with dichotomized pen-week (at least one pig with score ≥1 vs. no pig with score ≥1) as outcome variable and pen nested in batch as random effect (random intercept): results of the models for each individual explanatory variable, the full model and the final model

Fattening	Individual models		Full model		Final model^a^	
Fixed effect	Estimate	*P*-value	Estimate	*P*-value	Estimate	*P*-value
Week	0.062	0.429	−0.091	0.037	−0.067	0.087
SG (CG = baseline)	−0.380	0.325	−0.390	0.313		
day/night temperature range	−0.119	0.027	−0.106	0.074	−0.119	0.027
average temperature			−0.108	0.259		
ammonia concentration	0.018	0.174	0.013	0.405		
air velocity	−0.522	0.729	1.162	0.479		

^a^random effect batch:pen (Intercept): variance 1.301

**Table 7:** Results of the linear mixed-effects model for the fattening period with log-transformed summary score per pen-week as outcome variable and pen nested in batch as random effect (random intercept): results of the models for each individual explanatory variable, the full model and the final model

Fattening	Individual models		Full model		Final model^a^	
Fixed effect	Estimate	*P*-value	Estimate	*P*-value	Estimate	*P-*value
Week	−0.008	0.759	0.059	0.006	−0.724	0.009
SG (CG = baseline)	−0.075	0.724	−0.082	0.681		
day/night temperature range	−0.061	0.032	−0.033	0.303		
average temperature	−0.046	0.278	−0.077	0.088		
ammonia concentration	0.016	0.007	0.017	0.015	0.016	0.007
air velocity	−0.061	0.935	0.939	0.237		

^a^random effect batch:pen (Intercept): variance 0.2492, residual variance 0.3337

The results of serology (see Additional files 4 and 5), reveal infections with MH in all batches and with PRRSV in the second and third batch. Performing a PRRSV-PCR, the NA-genotype was found in two of eight pool samples of batch 2 at the end of the fattening period as well as in all eight pools of batch 3 at the beginning of the fattening period. The sequencing of the ORF7 region of the PRRSV strain detected in the third batch resulted in a homology of 99% with the PRRSV-NA vaccine strain. The sequencing of a strain from the second batch was not successful due to a low threshold cycle (Ct)-value. PCV2 was detected by PCR in two out of eight sample pools of the first and the fourth batch at the beginning of the fattening period.

The average daily straw- and feed consumption per pig and the average daily weight gain per pig are shown in Table 8. The average daily straw consumption in the SG was 3.5 g/pig (SD = 1.1) during the rearing period and 31.9 g/pig (SD = 7.7) during the fattening period. Comparing the average daily weight gain (SG = 809 g, SD = 58); CG = 800 g, SD = 74) during fattening and the average daily feed consumption per pig (SG = 2.1 kg, SD = 0.3; CG = 2.0 kg, SD = 0.3) of the SG and CG no significant differences were detected in the linear mixed-effects models (Table 9). In the SG, feed consumption and straw consumption in fattening were negatively but not significantly correlated.The mean values, minimum ﻿values, maximum values and the standard deviatio﻿ns (SD) of the measured temperatures, ammonia concentrations and air velocities as well as of the calculated day/night temperature ranges are presented in Tables 10 and 11 for each batch and period.

**Table 8:** Weight gain, straw consumption and feed consumption per pig and day during the rearing and fattening period

Batch	Group	Rearing period			Fattening period			
		duration [days]	weight gain (pig/day) [g]	straw consumption (pig/day) [g]	average duration [days]	weight gain (pig/day) [g]	straw consumption (pig/day) [g]	feed consumption (pig/day) [kg]
1	SG	61	537	3.3	99.3	827	32.7	2.0
1	CG	61	502	-	101.1	835	–	2.1
2	SG	38	466	3.0	115.2	849	25.0	2.0
2	CG	38	458	–	117.3	800	–	1.9
3	SG	43	446	4.8	123.9	737	36.7	1.9
3	CG	43	412	–	126.1	702	–	1.7
4	SG	50	639	3.7	91.4	826	25.5	2.4
4	CG	50	638	–	91.0	835	–	2.3

**Table 9:** Results of the four linear mixed-effects models: with treatment group as explanatory variable and 1) average daily weight gain per pig (ADG) in nursery, 2) ADG in fattening and 3) feed consumption in the fattening as outcome, as well as 4) straw consumption as explanatory variable and feed consumption in fattening as outcome; each model with batch as random effect (random intercept)

	Nursery		Fattening	
Outcome ADG				
Fixed effect:	Estimate	*P*-value	Estimate	*P*-value
SG (CG = baseline)	−19.350	0.113	−16.560	0.301
Random effect:	Variance	Residual variance	Variance	Residual variance
Batch	8345	1117	2720	4030
Outcome feed consumption				
Fixed effect:			Estimate	*P*-value
SG (CG = baseline)			−0.079	0.209
Random effect:			Variance	Residual variance
Batch			0.056	0.030
Fixed effect:			Estimate	*P*-value
Straw consumption			−0.012	0.143
Random effect:			Variance	Residual variance
Batch			0.022	0.038

**Table 10:** Air temperature and night/day temperature range during the rearing period (R) and the fattening period (F)

Batch	Period	Air temperature [C°]					Day/night temperature range [C°]				
		n	mean	min	max	SD	n	mean	min	max	SD
1	R	6337	25.91	22.60	35.30	2.23	66	3.62	0.7	8.15	2.06
1	F	6117	22.79	18.40	29.15	1.67	65	3.68	1.20	7.10	1.36
2	R	3595	24.35	21.90	28.90	1.48	42	0.73	0.40	1.35	0.22
2	F	8058	21.24	16.4	29.00	2.21	85	2.12	0.90	11.15	1.52
3	R	4080	23.59	18.30	27.50	1.20	43	1.06	0.45	4.65	0.84
3	F	4057	20.17	17.05	26.30	1.58	43	3.43	1.00	7.95	1.89
4	R	4697	24.64	21.20	29.10	1.34	50	1.71	0.50	5.70	1.25
4	F	7811	23.60	19.00	34.50	2.49	82	4.17	0.90	11.10	2.44

**Table 11:** Ammonia content and air velocity during the rearing period (R) and the fattening period (F) measured at weekly intervals at four (rearing) or six (fattening) locations in the unit

Batch	Period	Ammonia [ppm]					Air velocity [m/s]				
		n	mean	min	max	SD	n	mean	min	max	SD
1	R	10	9.65	0.00	20.50	7.82	10	0.08	0.05	0.12	0.02
1	F	8	5.58	0.00	10.00	3.94	9	0.18	0.10	0.55	0.14
2	R	6	6.96	4.25	8.50	1.54	6	0.05	0.04	0.08	0.02
2	F	13	22.50	6.83	43.67	11.00	12	0.09	0.06	0.12	0.02
3	R	7	9.39	6.00	14.50	3.57	6	0.05	0.04	0.07	0.01
3	F	12	10.57	5.17	15,83	3.81	12	0.10	0.05	0.19	0.04
4	R	6	9.04	2.75	19.5	5.54	6	0.07	0.05	0.10	0.02
4	F	8	6.29	1.30	11.10	2.89	9	0.15	0.09	0.21	0.04

## Discussion

In this study the occurrence of tail biting was mainly focused on four pens with 15 affected pigs in batch 2, probably triggered by a failure in the ventilation system. Another outbreak of tail biting was registered in one pen during the rearing period of batch 4 with five affected pigs without any apparent cause. Thus, the prevalence of tail biting was much lower than expected from farm history and allows no statistically substantiated evaluation of the FT regarding the occurrence of tail biting in the SG and CG.

Nevertheless, in line with previous studies providing straw in racks [12, 16] the FT was not able to totally prevent tail biting under the conventional housing conditions in this study. A reason for this could be the quantity of straw provided by the FT, which mainly influences the time during which pigs are occupied by foraging. Jensen et al. [17] figured out that an amount of 250 g straw per pig and day was the point where pigs stopped increasing the oral manipulation of straw. In another study 390 g straw per pig and day were necessary to achieve no further reduction in abnormal behaviour towards pen mates [18]. In contrast to our study, these studies were performed in pens with partly solid concrete floor. Providing comparable amounts of straw in the present study was not possible due to the fully slatted concrete floor and the liquid manure system. However, small amounts of straw are more effective in preventing tail biting in conventionally housed pigs than non-modifiable enrichment objects or barren environment [12, 16, 19].

Furthermore, the results suggest that a higher straw consumption could reveal a lower feed intake. Assessing this negative correlation, we should keep in mind that the straw consumption per pig was very low and we were not able to define the amount of straw, which fell through the slats and was not eaten by the pigs. Additionally, we found no significant differences in feed consumption and weight gain on comparing the SG and CG.

Bulens et al. [14] offered straw in four different types of application (Funbar, MIK Toy, rack and straw feeder) and found no difference in growth either. In contrast, other studies [16, 20, 21] showed that feed consumption and daily weight gain were higher in straw bedded systems. However, in straw bedded housing systems the amount of digested straw is difficult to measure and pigs are able to control their microenvironment [20] influencing the demand for energy. Therefore, the results are not comparable.

An explanation for the differences between batches concerning straw consumption could be that it was impossible to achieve an equally sized gap of all foraging towers by using the same adjustment due to the hand manufacturing of the foraging towers. Furthermore, some containers seemed to be easier to move than others so that the farmer had to regulate the adjustment based on his own estimation in order to try to provide the same amount of straw to all pigs of the treatment group.

Despite the low prevalence of tail biting, bite marks were frequently detected in all batches and were nearly equally distributed in both groups. The most accepted description of tail biting in literature by Taylor et al. [22] (“Two-stage tail-biting”) differentiates between a “pre-damage stage” and a “damage stage”. The “pre-damage stage” is suggested to be a precursor of tail biting and is considered as explorative “tail-in-mouth” behaviour without causing any visible damage to the skin. The “damage stage” begins if the tail starts bleeding due to dental manipulation [22, 23]. In contrast to this definition the prevalence and the distribution of bite marks in the present study indicate that these small skin lesions are a consequence of exploratory behaviour, which do not necessarily result in tail biting, at least in pigs with docked tails.

Furthermore, climatic conditions have often been linked to the physical comfort of pigs and seem to have to an influence on the occurrence of tail lesions in this study.

This is in accordance with the evaluation by Dutch pig farmers, who identified climate as the main risk factor for tail biting [24]. The impact of air velocity on the probability that a pen-week had at least one pig with score ≥1, shown in this study, is in line with Scheepens et al. [25]. In their study, pigs showed significantly more exploratory and agonistic behaviour on penmates in periods of draught, which were produced with cold air and a high air velocity [25]. Nevertheless, interpreting the measured values for air velocity in our study, we should keep in mind that these values might be subject to strong variation, represent only one point of time per week and might be influenced by movements of the animals as well as the observer. In accordance with our results, temperature changes, have previously been associated to the occurrence of tail biting [26]. However, the average temperature in the nursery period, which was negatively associated with the summary score in this study, had a contrary effect in the study of Smulders et al. [27]. Here a high temperature in the nursery was the most important factor influencing the occurrence of tail biting [27]. Regarding these results, we have to consider, that too high temperatures as well as too low temperatures have a negative impact on the pig’s physical comfort. The outbreak of tail biting during the fattening period in batch 2 was probably caused by a failure in the ventilation system, which is the most important factor in reduced air quality [28] and temperature changes.

Pigs are exposed to particulate matter and gases, such as ammonia (NH_3_), carbon dioxide (CO_2_), hydrogen sulfide (H_2_S), methane (CH_4_) and nitrous oxide (N_2_O) [29]. Particulate matter and ammonia are known to have an impact on animal health and behaviour by adversely influencing the mucosal clearance system and irritation of the respiratory epithelia [28, 29], whereas carbon dioxide and hydrogen sulfide (in usually measured concentrations) are less harmful to animal health [28] and methane and nitrous oxide are related to global climate changes [30].

O’Connor et al. [31] assumed that atmospheric ammonia concentrations of 20 ppm had a negative impact on the pigs’ behaviour and physical comfort, while Jones et al. [32] showed that pigs already avoided ammonia concentrations of 10 ppm. Former attempts to induce tail biting by worsening the air quality were only partially successful [26, 33]. In our study, ammonia concentration was positively associated with the summary score (*p* = 0.007).

In addition to increasing ammonia contents a causal relationship of a risen air temperature should not be neglected. Geers et al. [34] suggested that inadequate temperature should be listed as a possible cause of tail biting. In their study tail biting was positively associated to an air temperature of 22—24 °C in pigs of 30—40 kg and to a temperature range of 16—18 °C in pigs weighing 40—50 kg. The pigs were housed on 40% partly slatted floors with floor heating at the beginning of the fattening period. With respect to this study the optimal air temperature range depends on the existing housing conditions. Lying, excreting and fouling behaviour are indicators for evaluating the pigs’ optimal temperature range [35]. In the absence of bedding, an air temperature of 20—16 °C for pigs of 40—50 kg and a temperature of 18—14 °C for pigs of 60—100 kg is recommended, the temperature reducing with increasing age of the pig [36]. In our study, especially during the week tail biting occurred in batch 2, the average temperature was far too high for the pigs with an assumed weight of approximately 55—65 kg per pig. Furthermore, pigs had been exposed to high temperature differences in the previous week. For practical reasons we did not record the behaviour of pigs.

D’Eath et al. [37] emphasize relationships between various known or suspected risk factors and the occurrence of tail biting. Among other potential risk factors, malfunction of the ventilation adversely affecting temperature and air quality might entail increasing activity levels and more pig directed exploratory behaviour because pigs compete for lying areas, which could improve their physical comfort. Damaging tail biting proceeds when other pigs tolerate tail manipulation.

Thus, the relationship between high ammonia levels, high air temperature and tail biting is probable in this case. Nevertheless, we have to consider that other parameters for air quality such as particulate matter, carbon dioxide, hydrogen sulfide and air humidity, not measured in this study play a role and might also have had an impact on tail biting.

Another potential risk factor for tail biting, identified by Taylor et al. [10], is the parameter health. In a study by Moinard et al. [9] tail biting was positively associated with respiratory disease. Performing a pathological examination, Munsterhjelm et al. [38] assume that most lung lesions were secondary to infected tail lesions, but disease could not be ruled out as a possible cause of tail biting. In order to assess respiratory diseases as a potential trigger in this study paired serum samples were analysed for the most prevalent pathogens causing primary infections of the respiratory tract in this age group and area – MH, PCV2 and PRRSV. Despite vaccinating the piglets against MH and PCV2 at the end of the suckling period increasing antibody titers against MH during the fattening period (all batches) and the detection of PCV2 by PCR (batches 1 and 4) revealed infections not causing clinical respiratory disease. The PRRS-NA vaccine strain (ATCC VR2332) and strains with a nucleotide identity of 98–100% in ORF 5 were frequently found in herds where only the sows had been vaccinated with Ingelvac® PRRS MLV [39]. The circulating PRRSV-NA strain in batch 2 probably originates from Ingelvac® PRRS MLV used in the sow herd. With regard to tail biting during the rearing period (batch 4), serum samples of this age group were not investigated as serology is unsuitable due to missing discrimination of possibly co-existing maternal antibodies.

## Conclusion

Due to the low prevalence of tail biting in all batches the effect of the FT tower could not conclusively be evaluated. Operating the FT with an average daily straw consumption of 3.5 g/pig (SD = 1.1) during the rearing period and 31.9 g/pig (SD = 7.7) during the fattening period did not affect the weight gain. Exploratory behaviour seems to cause bite marks, which do not result in tail biting. The main outbreak of tail biting was probably triggered by a failure of the ventilation system, which resulted in a number of climatic and air quality changes including higher ammonia concentrations and sudden temperature changes.

## Additional files

Additional file 1: a: Bite marks (score 1) and tail wounds (score ≥ 2) related to the ammonia content (NH_3)_ in the unit of batch 1 (w = week). b: Bite marks (score 1) and tail wounds (score ≥ 2) related to the average temperature and the highest day/night temperature range per week in the unit of batch 1 (w = week). (PDF 76 kb)

Additional file 2: a: Bite marks (score 1) and tail wounds (score ≥ 2) related to the ammonia content (NH_3)_ in the units of batch 3 (w = week). b: Bite marks (score 1) and tail wounds (score ≥ 2) related to the average temperature and the highest day/night temperature range per week in the unit of batch 3 (w = week). (PDF 54 kb)

Additional file 3: a: Bite marks (score 1) and tail wounds (score ≥ 2) related to the ammonia content (NH_3)_ in the units of batch 4 (w = week). b: Bite marks (score 1) and tail wounds (score ≥ 2) related to the average temperature and the highest day/night temperature range per week in the unit of batch 4 (w = week). (PDF 66 kb)

Additional file 4: Results of the IDEXX PRRS X3® ELISA. (PDF 289 kb)

Additional file 5: Results of the IDEXX M. hyo® ELISA. (PDF 334 kb)

## Abbreviations

ADG: Average daily weight gain
CG: Control group
e. g: exempli gratia (for example)
EFSA: European Food Safety Authority
ELISA: Enzyme linked immunosorbent assay
F: Fattening period
FT: Foraging-tower
ME: metabolizable energy
MH: *Mycoplasma hyopneumoniae*
MLV: Modified live vaccine
NA: North American
PCR: Polymerase chain reaction
PCV2: Porcine Circovirus Type 2
PRRS: Porcine reproductive and respiratory syndrome
PRRSV: Porcine reproductive and respiratory syndrome virus
R: Rearing period
SD: Standard deviation
SG: Straw group
SIV: Swine Influenza Virus
