# Supplementary figures and images for: Provision of straw by a foraging tower –effect on tail biting in weaners and fattening pigs
Source: Porcine Health Manag. 2017 Mar 16;3:4. doi: 10.1186/s40813-017-0052-7 (PMC5382410; doi:10.1186/s40813-017-0052-7)

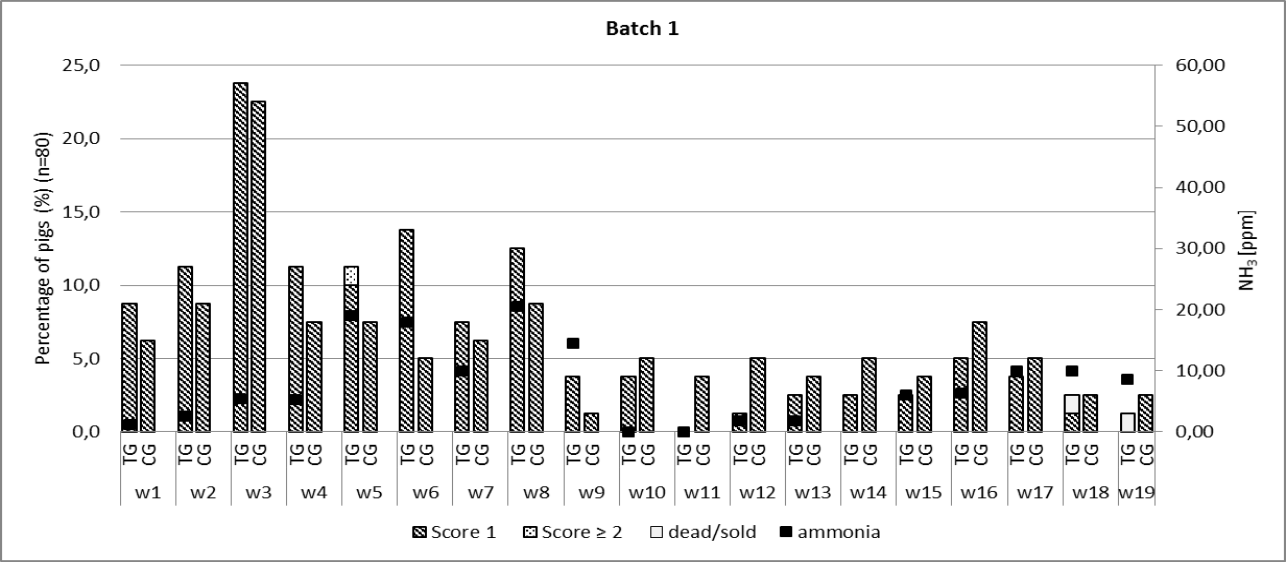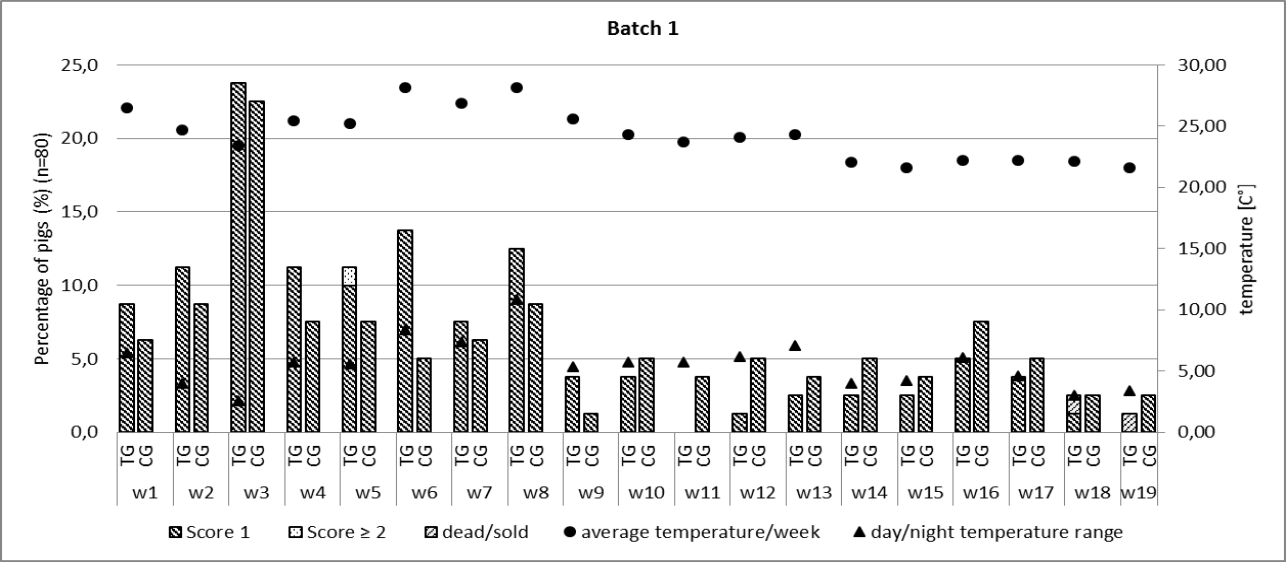

Supplement: Supplementary file 1 — a: Bite marks (score 1) and tail wounds (score ≥ 2) related to the ammonia content (NH3) in the unit of batch 1 (w = week). b: Bite marks (score 1) and tail wounds (score ≥ 2) related to the average temperature and the highest day/night temperature range per week in the unit of batch 1 (w = week). (PDF 76 kb) [file 40813_2017_52_MOESM1_ESM.pdf]

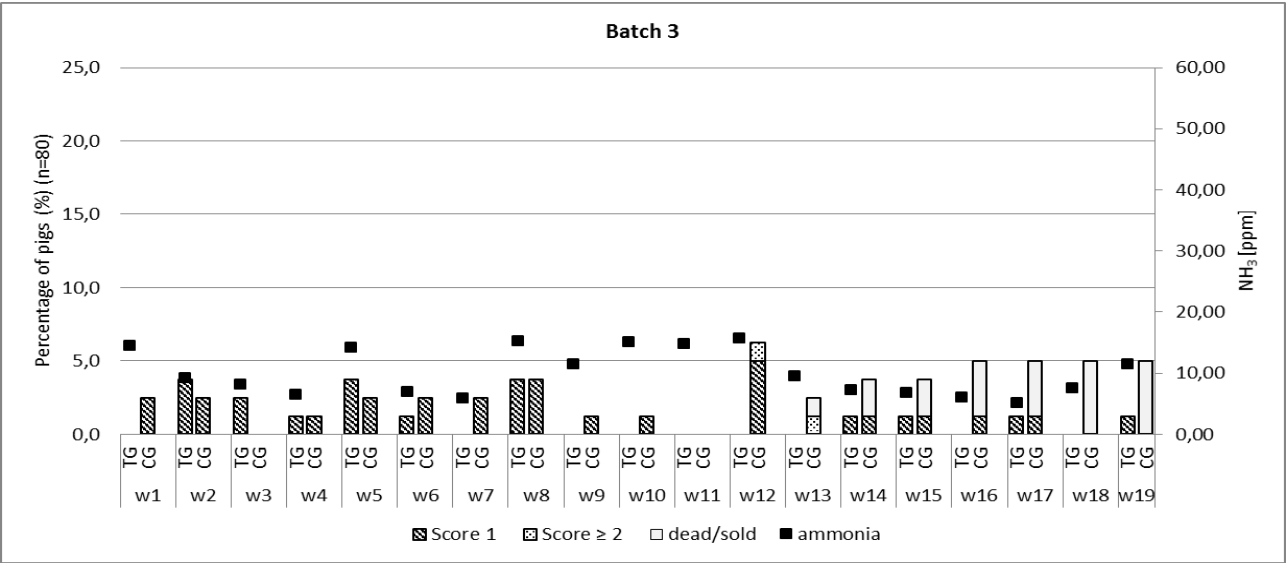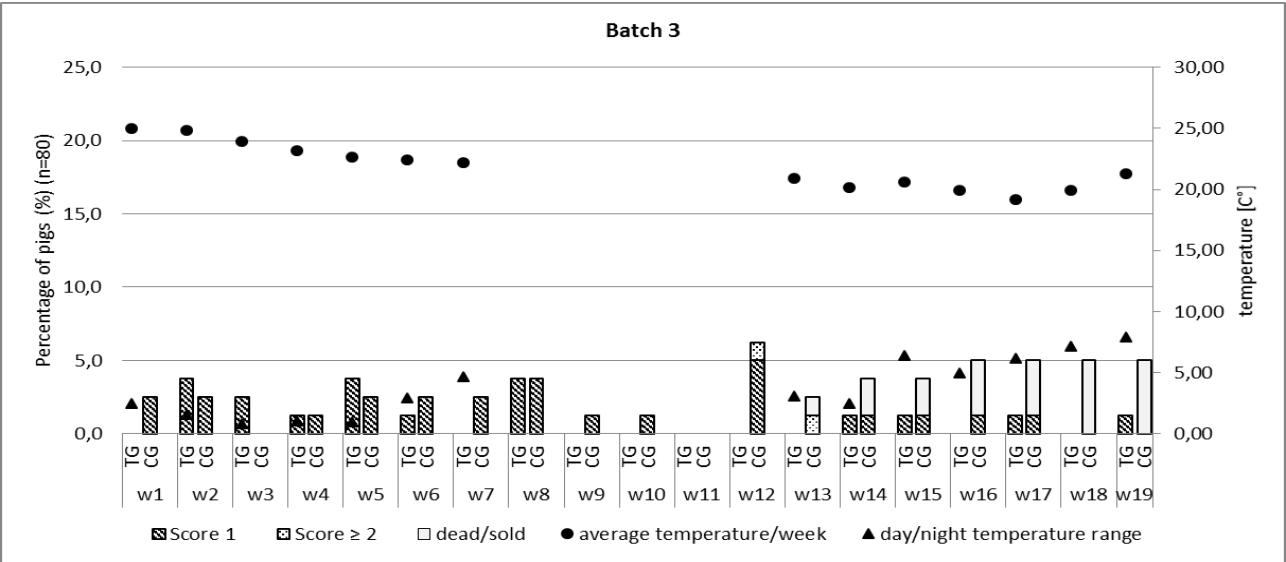

Supplement: Supplementary file 2 — a: Bite marks (score 1) and tail wounds (score ≥ 2) related to the ammonia content (NH3) in the units of batch 3 (w = week). b: Bite marks (score 1) and tail wounds (score ≥ 2) related to the average temperature and the highest day/night temperature range per week in the unit of batch 3 (w = week). (PDF 54 kb) [file 40813_2017_52_MOESM2_ESM.pdf]

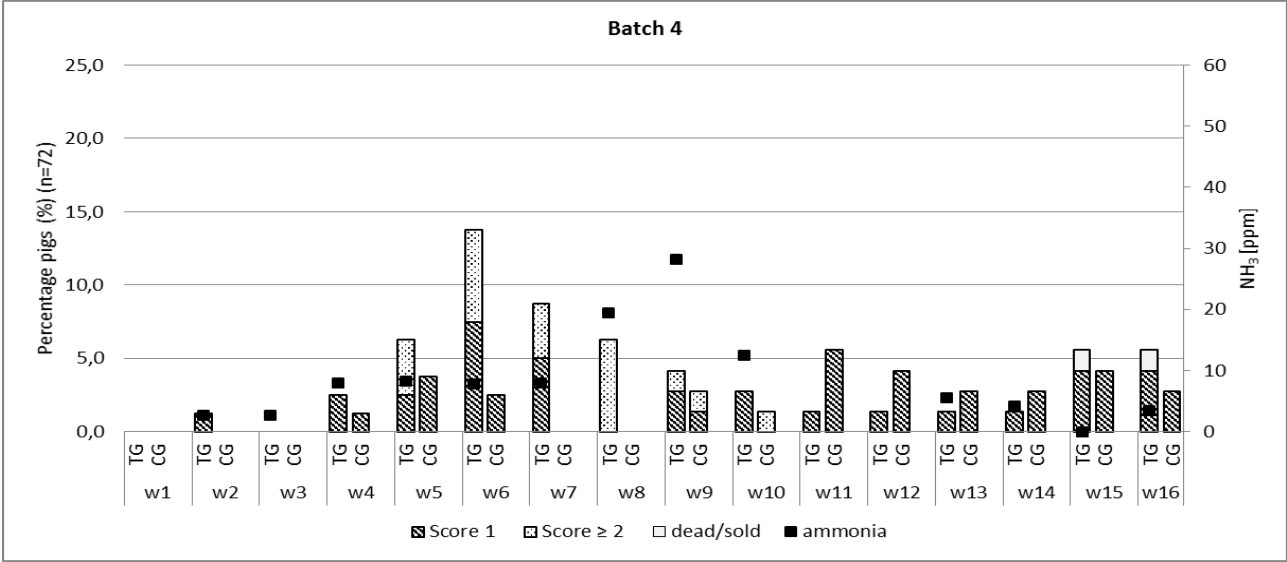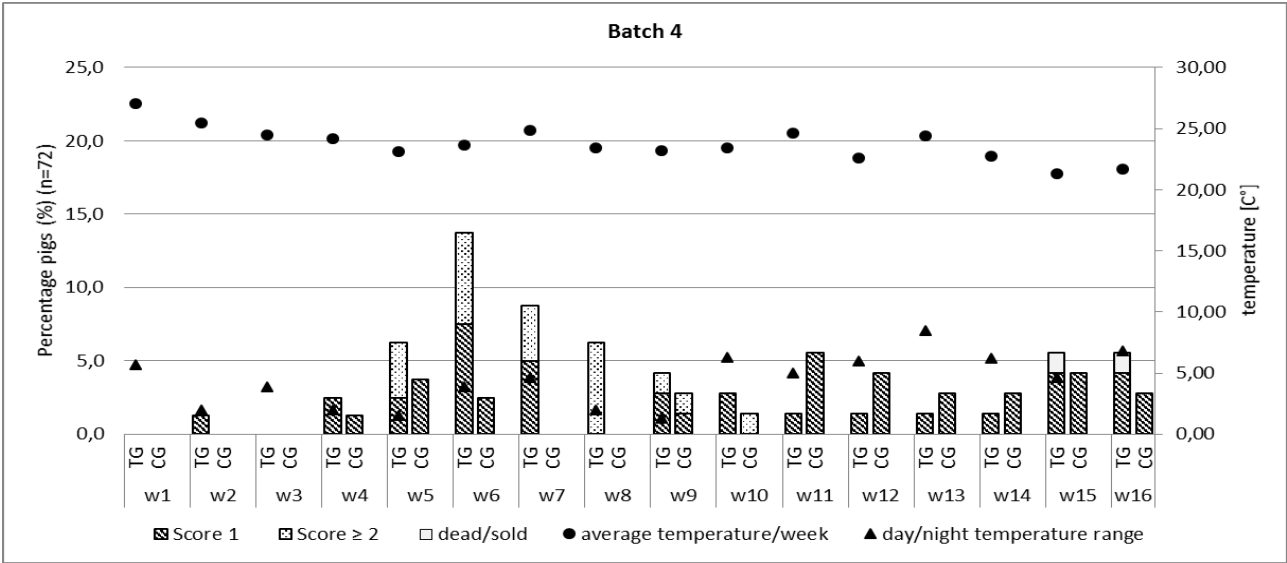

Supplement: Supplementary file 3 — a: Bite marks (score 1) and tail wounds (score ≥ 2) related to the ammonia content (NH3) in the units of batch 4 (w = week). b: Bite marks (score 1) and tail wounds (score ≥ 2) related to the average temperature and the highest day/night temperature range per week in the unit of batch 4 (w = week). (PDF 66 kb) [file 40813_2017_52_MOESM3_ESM.pdf]

Results IDEXX PRRS X3® ELISA

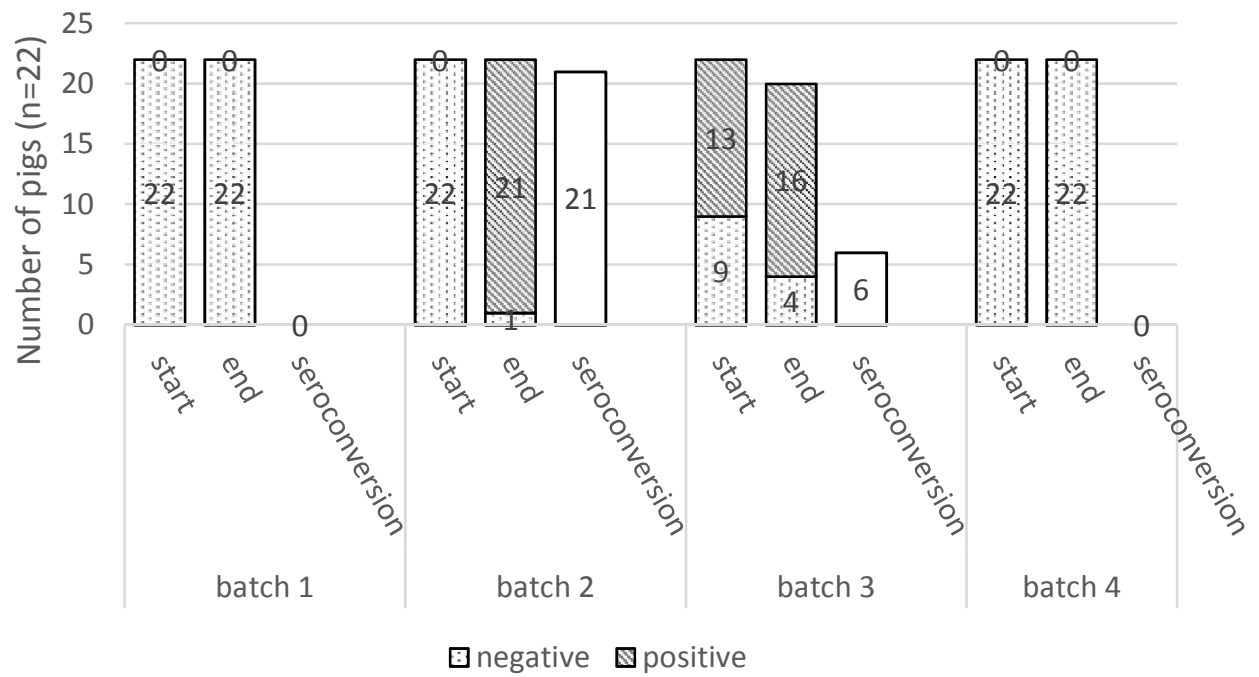

Supplement: Supplementary file 4 — Results of the IDEXX PRRS X3® ELISA. (PDF 289 kb) [file 40813_2017_52_MOESM4_ESM.pdf]

### Results IDEXX M. hyo<sup>®</sup> ELISA

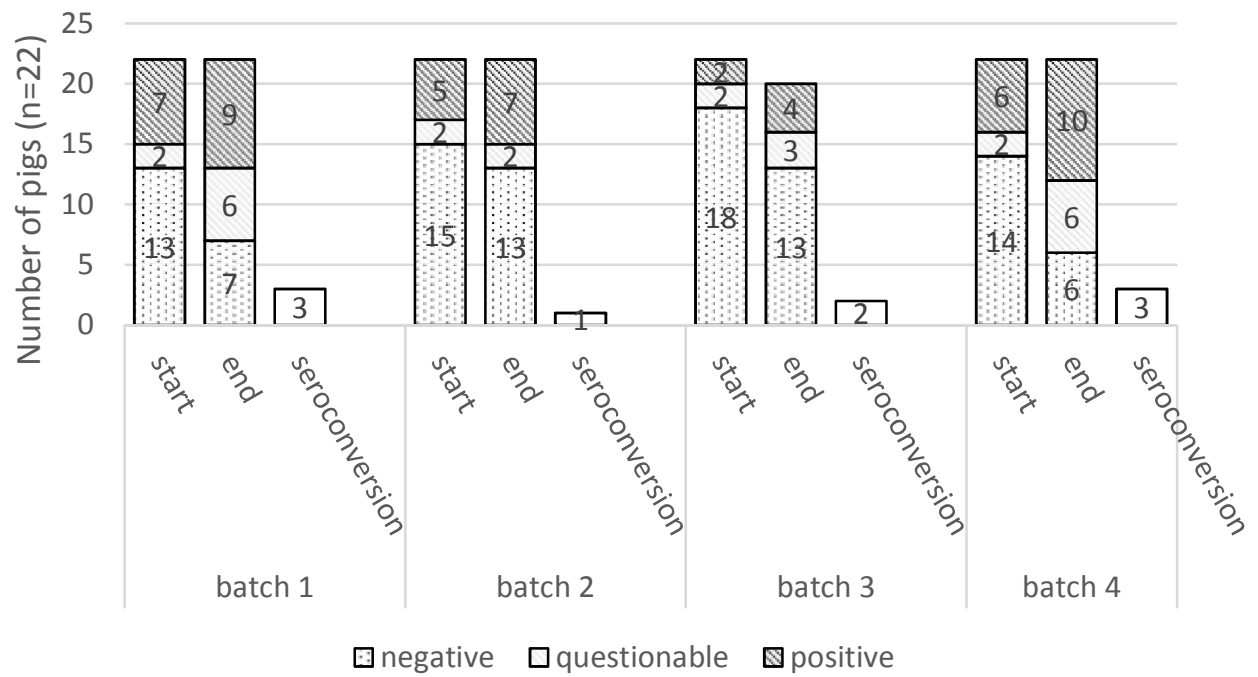

Supplement: Supplementary file 5 — Results of the IDEXX M. hyo® ELISA. (PDF 334 kb) [file 40813_2017_52_MOESM5_ESM.pdf]
